# Glyceraldehyde-3-phosphate dehydrogenase (GAPDH) moonlights as an adhesin in *Mycoplasma hyorhinis* adhesion to epithelial cells as well as a plasminogen receptor mediating extracellular matrix degradation

**DOI:** 10.1186/s13567-021-00952-8

**Published:** 2021-06-03

**Authors:** Jia Wang, Yao Li, Longji Pan, Jun Li, Yanfei Yu, Beibei Liu, Muhammad Zubair, Yanna Wei, Bala Pillay, Ademola Olufolahan Olaniran, Thamsanqa E. Chiliza, Guoqing Shao, Zhixin Feng, Qiyan Xiong

**Affiliations:** 1 Institute of Veterinary Medicine, Key Laboratory of Veterinary Biological Engineering and Technology, Ministry of Agriculture, Jiangsu Academy of Agricultural Sciences, Nanjing, China; 2 College of Agriculture, Engineering & Science, University of KwaZulu-Natal, Durban, South Africa; 3 School of Life Sciences, Jiangsu University, Zhenjiang, China; 4 College of Veterinary Medicine, Hunan Agricultural University, Changsha, China; 5 School of Food and Biological Engineering, Jiangsu University, Zhenjiang, China

**Keywords:** *M. hyorhinis*, GAPDH, Adhesion, Plasminogen, Extracellular matrix

## Abstract

*Mycoplasma hyorhinis* infects pigs causing polyserositis and polyarthritis, and has also been reported in a variety of human tumor tissues. The occurrence of disease is often linked with the systemic invasion of the pathogen. Glyceraldehyde-3-Phosphate Dehydrogenase (GAPDH), one of the key enzymes of glycolysis, was reported as a surface multifunctional molecule in several bacteria. Here, we investigated whether GAPDH could manifest binary functions; as an adhesin to promote colonization as well as a plasminogen receptor functioning in extracellular matrix (ECM) degradation to promote systemic invasion. The surface localization of GAPDH was observed in *M. hyorhinis* with flow cytometry and colony blot analysis. Recombinant GAPDH (rGAPDH) was found to be able to bind porcine-derived PK-15 and human-derived NCI-H292 cells. The incubation with anti-GAPDH antibody significantly decreased the adherence of *M. hyorhinis* to both cell lines. To investigate its function in recruiting plasminogen, firstly, the interaction between rGAPDH and plasminogen was demonstrated by ELISA and Far-Western blot assay. The activation of the rGAPDH-bound plasminogen into plasmin was proved by using a chromogenic substrate, and furtherly confirmed to degrade extracellular matrix by using a reconstituted ECM. Finally, the ability of rGAPDH to bind different ECM components was demonstrated, including fibronectin, laminin, collagen type IV and vitronectin. Collectively, our data imply GAPDH as an important adhesion factor of *M. hyrohinis* and a receptor for hijacking host plasminogen to degrade ECM. The multifunction of GAPDH to bind both plasminogen and ECM components is believed to increase the targeting of proteolysis and facilitate the dissemination of *M. hyorhinis*.

**Supplementary Information:**

The online version contains supplementary material available at 10.1186/s13567-021-00952-8.

## Introduction

The cell wall-less members of the *Mycoplasma* genus are commensal, opportunistic or pathogenic bacteria that can colonize in humans, animals and plants. Various *Mycoplasma* species are considered pathogenic to swine, including *Mycoplasma hyopneumoniae*, *Mycoplasma hyorhinis, Mycoplasma suis* and *Mycoplasma hyosynoviae* [1]. *M. hyorhinis* is ubiquitous in the pig population and can be found in the respiratory tract of both healthy animals and those showing clinical signs of *M. hyorhinis* infection. Most colonized pigs show no apparent clinical manifestation of disease. The occurrence of disease is often related to the systemic invasion of the pathogen. Clinical signs vary greatly from server polyserositis, arthritis and lameness, to mild otitis or conjunctivitis [1–5]. In addition to being a pathogen of pigs, *M. hyorhinis* has also been reported to be linked with human cancer [2, 3]. *M. hyorhinis* has been detected in cancerous tissues from gastric, esophageal, lung, breast, glioma and colon cancers by polymerase chain reaction (PCR) or immunohistochemical staining (IHC) [2]. Antibodies to *M. hyorhinis*, specifically targeted to the P37 protein, have also been found to bind circulating tumor cells (CTCs) in patients with hepatocellular carcinoma [4, 5].

Adhesion factors play a vital role in the process of mycoplasma infection and pathogenicity. At present, there are very limited research reports on the adhesion factors of *M. hyorhinis*. To date, only two adhesion molecules have been identified, these include variable lipoprotein family (Vlp) [6] and the tumor-associated lipoprotein P37 [7]. However other adhesins, colonization factors and pathogenicity mechanisms are yet to be uncovered. As described for several other species of mycoplasma, many endogenous proteins are also expressed on the surface of the cell membrane due to unknown mechanism, and exert the function as adhesion molecules [8–11]. Among them, the enzymes in the glycolysis pathway are the most common proteins. Due to the absence of a TCA cycle, mycoplasmas often, but not exclusively derive ATP from glycolysis. Its key enzymes such as enolase, GAPDH, fructose-1,6-diphosphate aldolase, pyruvate dehydrogenase have all been reported to have cell adhesion functions and are processed on the cell surface [8, 12–15]. In addition, these proteins are often found to bind the components of the ECM [16, 17], extracellular actin [18] and plasminogen [13, 19, 20].

On the other hand, a variety of mycoplasmas, including *M. hyorhinis*, are invasive bacteria [21–23]. A common feature of invasive processes is the degradation of ECM or basement membrane (BM, specialized ECM), which is required for invasive cells to migrate into adjacent tissues or into circulation [24]. Various invasive bacterial pathogens express plasminogen receptors (PlgR) that immobilize plasminogen on its surface. The bound plasminogen is converted to plasmin by tissue plasminogen activator (tPA) or urokinase plasminogen activator (uPA), and initiate a proteolytic cascade that leads to damage to these tissue barriers [24, 25]. Furthermore, bacterial adherence to ECM (including BM) initiated bacterial uptake via the endosomal pathway [26, 27] and directs bacterium-induced proteolytic activity onto ECM, which facilitates bacterial dissemination through ECM [24, 28].

GAPDH, which catalyzes the conversion of glyceraldehyde 3-phosphate to 1,3-diphosphoglycerate, is a typical moonlighting enzyme. Surface expression and adhesive function have been confirmed for an increasing number of bacteria, including *Streptococcus* species [29], *Erysipelothrix rhusiopathiae* [30], *Lactobacillus reuteri* [31], *Mycoplasma pneumoniae* [12, 16, 32]. The GAPDH of *M. pneumoniae* has been proved to bind different types of human cells [12, 32] and interact with human plasminogen, vitronectin, fibronectin and fibrinogen [16, 32]. The role of *Mycoplasma hyopneumoniae* GAPDH in adhesion was also strongly implicated by its interaction with actin and other cell-surface associated components [18].

In the present study, we identified the participation of *M. hyorhinis* GAPDH in cytoadherence to swine and human cells, interaction with ECM, and its role in hijacking plasminogen/plasmin system to degrade ECM components.

## Materials and methods

### Bacterial strains, cell line, plasmids and cultivation

*M. hyorhinis* strain HEF16 was isolated in our lab from a pig showing typical clinical signs, and grown in KM2 medium (Jiangsu Academy of Agricultural Sciences, China). *Escherichia coli* strains DH5α and BL21 (DE3) were cultured in Luria–Bertani (LB) broth or on solid media containing 1.5% agarose supplemented with 30 μg/mL of kanamycin. The pET-28a ( +) expression vector was obtained from Novagen (Merck, Germany).

PK15 cells, a porcine epithelial cell line derived from a normal pig kidney, and NCI-H292 cells, a human airway epithelial cell line derived from a pulmonary mucoepidermoid carcinoma were purchased from the American Type Culture Collection (ATCC). RPMI 1640 + 10% FBS medium was used for cells culture. The cells were maintained in a humidified air with provision of 5% CO2.

### Cloning and expression of the GAPDH gene

The entire GAPDH gene was generated synthetically based on the sequence of strain HUB-1(GenBank, CP002170.1). The sequence was optimized with *E. coli*-preferred codons and two TGA codons were mutated into TGG. The gene was inserted into the pET-28a ( +) vector between the *Xho*I and *Bam*HI cleavage sites. The recombinant plasmids were introduced into *E. coli* BL21 by chemical transformation (BL21 Chemically Competent Cells, Sigma, USA), identified by PCR with T7 promoter and T7 terminator primers and verified by DNA sequencing (GenScript, China). *E. coli* cells at log phase were treated with isopropyl-beta-D-thiogalactopyranoside (IPTG) with a final concentration of 1 mM for 6 h at 37 ℃ to induce expression. Recombinant protein was purified by nickel affinity chromatography (GenScript, China), dialyzed, freeze-dried and stored at −80 °C before use. Protein concentration was measured by bicinchoninic acid (BCA) assay kit (Beyotime Biotechnology, China). For identification, whole cell lysate of *E. coli* BL21 carrying recombinant vector pET-28a-*gapdh* before and after induction by IPTG and purified rGAPDH protein were subjected to 12% SDS-PAGE and transferred to a polyvinylidene fluoride (PVDF) membrane (Millipore, Germany). After blocking with 5% skim milk in TBST buffer (20 mM Tris–HCl (pH 7.6), 150 mM NaCl and 0.1% Tween-20), the membrane was incubated with mouse anti-His-tag monoclonal antibody (1:1000, Boster, China), followed by horseradish peroxidase (HRP)-conjugated goat anti-mouse IgG (1:10,000, Boster, China). Finally, filters were developed with Electro-Chemi-Luminescence (ECL) substrate using a ChemiDoc XRS + system (Bio-Rad, USA).

### Preparation of polyclonal antibody against rGAPDH

Polyclonal antibodies were raised against rGAPDH by subcutaneously immunizing two 1-month-old New Zealand White rabbits which were obtained from a contract farm. Each rabbit was immunized three times with 1 mg of rGAPDH emulsified in Freund’s adjuvant (1:1, v/v, Sigma, USA) at 2-week intervals. Antisera were collected at 1 week after the third immunization, and titers were determined by ELISA. The reactivity and specificity of the prepared polyclonal antibody was assessed by Western blotting (Additional file 1).

### Detection of surface exposed GAPDH in *Mycoplasma hyorhinis* cells

Flow cytometry analysis was used to detect if GAPDH was located on the surface of *M. hyorhinis*. In brief, *M. hyorhinis* (10^8^ color change unit (CCU)) were incubated with anti-rGAPDH serum at a 1:100 dilution (preimmune rabbit serum was used as negative control) for 1 h at 37 °C. The blank control was incubated with PBS instead of antibody. *M. hyorhinis* cells were stained with fluorescein isothiocyanate (FITC)-conjugated goat anti-rabbit IgG at a 1:500 dilutions (Boster, China) for 1 h at 37 °C. The fluorescence intensity was detected using flow cytometer (BD Accuri® C6). The level of mean fluorescence intensity (MFI) of *M. hyorhinis* incubated with anti-rGAPDH serum was expressed as the percentage of that incubated with preimmune serum.

Alternatively, the colony blot technique was used to detect if GAPDH displayed at the surface of *M. hyorhinis* colonies. PVDF membranes were gently placed on mycoplasma colonies on the surface of agar plates. After 5 min, filters were removed, blocked for 1 h at 37 °C with TBST buffer containing 5% skim milk, and incubated overnight at 4 °C in TBST containing 5% skim milk and anti-rGAPDH serum (1:1000 dilution). Filters were washed four times with TBST with an interval of 15 min and treated with HRP-conjugated goat anti-rabbit IgG (1:10 000; Boster, China) for 1 h at 37 °C. Finally, filters were developed with ECL substrate using the ChemiDoc XRS + system. Preimmune serum was used as a negative control instead of anti-rGAPDH serum.

### Binding ability of rGAPDH to human and swine airway epithelial cells

PK-15 or NCI-H292 cells were propagated in a 96-well cell plates for 24–36 h. The original medium was removed, and 100 μg of rGAPDH in medium was added and incubated at 37 °C for 2 h after washing the cells thrice with PBS. Cells incubated with BSA were used as control. Unbound proteins were washed with PBS. The cells were then fixed with cold ethanol for 30 min at 4 °C, and blocked with PBS containing 5% BSA for 1 h at 37 °C. The adherence was evaluated by adding anti-rGAPDH monoclonal antibody (1:1000 dilution) followed by Cy3-conjugated goat anti-rabbit IgG (1:1000; Beyotime Biotechnology, China). The cell nuclei were stained with Hoechest 33,342 (Beyotime Biotechnology, China). After washing, the immunofluorescence was detected under a fluorescence microscope (Zeiss, Germany).

Micro titer plate adhesion assay (MPAA) was conducted in order to quantitatively detect the binding between rGAPDH and cell membrane protein. For this, cell membrane proteins were prepared by a commercial Membrane and Cytosol Protein Extraction Kit according to the manufacturer’s instructions (Tiangen Biotech, China). A flat-bottom 96-well ELISA plate was coated with 100 μL cell membrane protein (10 μg/mL) overnight at 4 °C. After blocking with 5% BSA, the plate was incubated for 2 h at 37 °C with 100 μL of different concentrations (from 1.5 to 100 μg/mL) of rGAPDH protein. PBS was used as control. Unbound proteins were removed by washing with PBST, and the adherence were evaluated by adding 100 μL of mouse anti-His-tag monoclonal antibody (1:5000), followed by 100 μL of HRP-conjugated goat anti-mouse IgG (1:10 000). After washing, the substrate containing 3,3’,5,5’-tetramethylbenzidine and H_2_O_2_-urea was added and the plates were incubated at 37 °C for 15 min. Then, H_2_SO_4_ was added to stop the reaction, absorbance was measured at 450 nm. For the adherence inhibition assay, 50 μg/mL of rGAPDH was mixed rabbit anti-rGAPDH serum (1:100 dilution) and added into the microtiter plate. Experiments were performed in triplicate.

### Adhesion inhibition of *Mycoplasma hyorhinis* to cells by anti-rGAPDH polyclonal antibodies

*Mycoplasma hyorhinis* cells (1 × 10^7^ CCU/mL) were washed three times with PBS and pre-incubated with polyclonal antibody raised against rGAPDH or preimmune sera (1:100 dilution) at 37 °C for 30 min. Bacteria suspended in RPMI-1640 medium were added to 96-well cell plates containing confluent PK-15 or NCI-H292 cells and incubated at 37 °C for 6 h. After washing with PBS to remove nonadherent mycoplasmas, the cells were digested with 0.25% trypsin at 37 °C for 5 min, and the CCU of mycoplasma were determined. The sample solution (100 μL) was added into 900 μL of medium, the serial ten-fold dilutions were made until a dilution of 10^–11^ was achieved. The cultures were incubated at 37 °C for 14 days. The highest dilution at which color changes was detected and recorded in CCU/mL. Experiments were performed in triplicate.

### Binding activity of rGAPDH to plasminogen

ELISA and Far-Western blotting were used to determine the binding ability of rGAPDH to plasminogen. For ELISA, 96-well micro titer plates were coated with 100 μL rGAPDH solution (30 μg/mL) overnight at 4 °C. After blocking with 5% BSA, the plate was incubated for 2 h at 37 °C with 100 μL of human plasminogen (10 μg/mL, Sigma, USA) or BSA. After washing with PBST, binding properties were determined by incubating with rabbit anti-plasminogen polyclonal antibody (1:2000 dilution; Boster, China) followed by HRP-conjugated goat anti-rabbit IgG (1:10 000 dilution). After washing, the substrate was added and the absorbance was measured at 450 nm.

For Far-Western blotting, a 20 μg sample of rGAPDH was separated by 12% SDS-PAGE and transferred to a PVDF membrane. After blocking with 5% skim milk, the membrane was incubated with 10 μg/mL plasminogen, followed by incubation with rabbit anti-plasminogen polyclonal antibody (1:1000 dilution) as the primary antibody, and HRP-conjugated goat anti-rabbit IgG (1:10 000 dilution) as the secondary antibody. Finally, the membrane was developed with ECL as described above. BSA was used as negative control.

### Plasminogen activation assay and the degradation of ECM

The 96-well micro titers plates were coated with 100 μL rGAPDH solution (30 μg/mL) overnight at 4 ℃. After blocking with 5% BSA, the plate was incubated for 2 h at 37 °C with 100 μL of human plasminogen (5 μg/mL, Sigma, USA) in the presence or absence of 200 mM lysine analogue, ε-aminocaproic acid (ε-ACA, Sigma, USA). After washing with PBS, 100 μL of tPA (200 ng/mL, Sigma, USA) was added and the plate was incubated for 2 h at 37 °C. The plasmin-specific substrate D-valyl-leucyl-lysine-p-nitroanilide dihydrochloride (Sigma, USA) was added at a final concentration of 0.4 mM. Plates were incubated overnight at 37 °C, and absorbance was read at 405 nm.

To visualize the ECM degradation by scanning electron microscope, the rGAPDH harboring polystyrene beads were used. Conjugation of rGAPDH protein and BSA with polystyrene beads of 1.1 μm mean particle size (Sigma, USA) was performed essentially as described by the manufacturer using the passive absorption procedure. In brief, 20 μL beads were incubated with 1 mL of rGAPDH or BSA solution of 1.5 mg/mL overnight at 4 °C, followed by blocking with BSA (5%). The binding of rGAPDH was detected by anti-His-tag antibody (Data not shown). After washing with PBS, 10 μg/mL plasminogen was added and incubated for 3 h at 37 °C, followed by 200 ng/mL tPA. After washing, the beads were resuspended in 1 mL PBS. Matrigel was diluted in ice-cold PBS (1:3) and pipetted on 3-μm filters in Transwell cell culture chamber inserts (Corning, USA). The Matrigel (Corning, USA), a BM preparation from mouse tumor, was allowed to settle at 4 °C for 30 min and was then gelled at 37 °C overnight. The Matrigel was rehydrated in 70 uL PBS for 1 h at 37 °C before proceeding with the degradation assay. The resuspended beads of 70 uL were added into the upper compartment of the Transwell whereas the lower compartment contained 700 uL PBS. The chambers were incubated at 37 °C for 40 h. The filters were gently washed with PBS and fixed with 2.5% glutaraldehyde, and exanimated in a Zeiss EVO-LS10 scanning electron microscope (Zeiss, Germany).

### Binding activity of rGAPDH to ECM

The 96-well micro titers plate were coated with 100 μL Matrigel, fibronectin, laminin, collagen IV, or BSA solution (10 μg/mL) overnight at 4 °C. After blocking with 5% BSA, the plate was incubated for 2 h at 37 °C with 100 μL of rGAPDH protein (12.5–100 μg/mL) or BSA. Unbound proteins were removed by washing with PBST, and the adherence was evaluated by adding 100 μL of mouse anti-His-tag monoclonal antibodies (1:1000), followed by 100 μL of HRP-conjugated goat anti-mouse IgG (1:10 000). After washing, the substrate was added and the absorbance was measured at 450 nm. The ELISA system was inverted for detecting the binding of rGAPDH to recombinant vitronectin (containing His-tag). rGAPDH or BSA protein of 10 μg/mL were coated, and then vitronectin (1.25–5 μg/mL) were added. The bound vitronectin was detected by rabbit anti-vitronectin monoclonal antibody (1:2000; Boster, China), followed by goat anti-rabbit IgG (1:10 000).

### Statistical analysis

Statistical difference was analyzed by one-way ANOVA. The *P*-values < 0.05 (*) were considered as statistically significant.

## Results

### Expression and purification of rGAPDH

**Figure 1:**
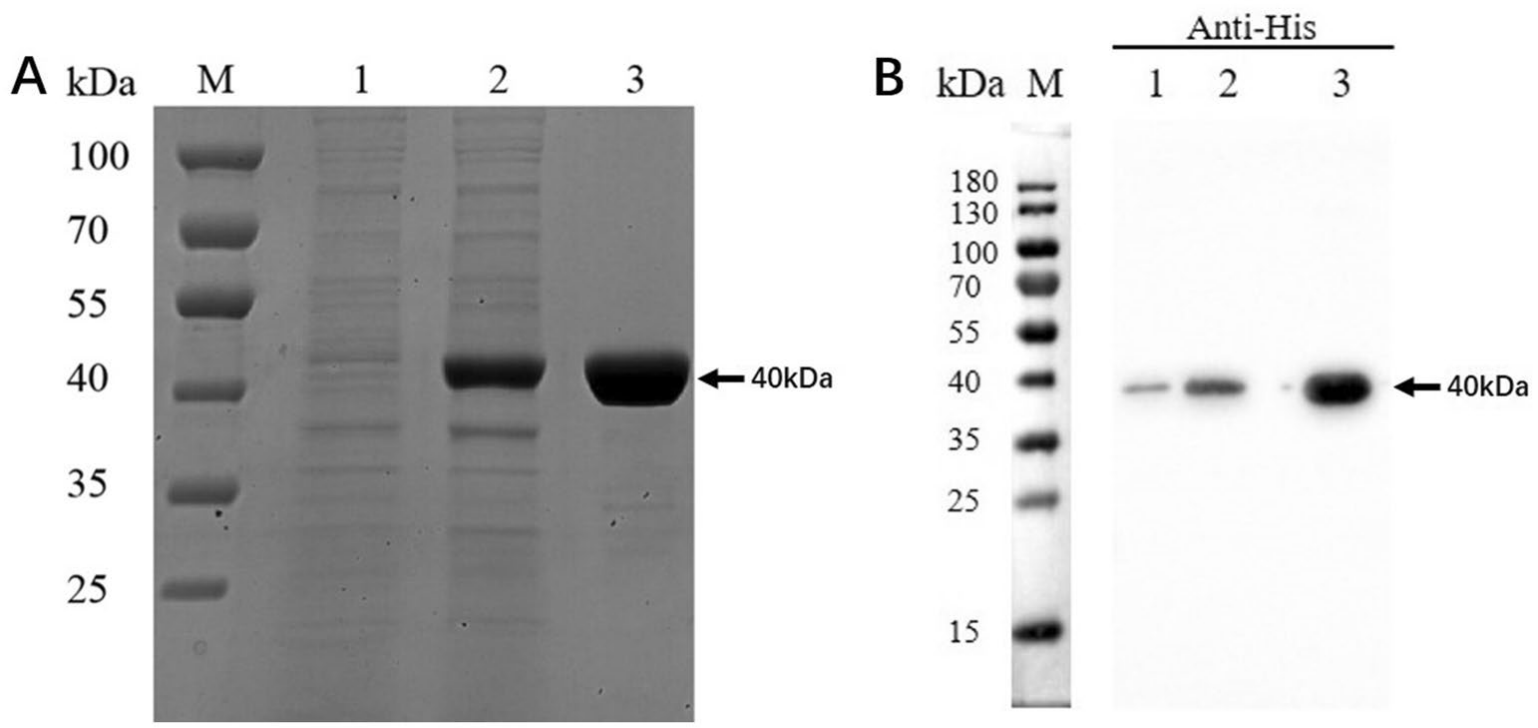
**Expression and purification of the rGAPDH**. **A** SDS-PAGE analysis of the rGAPDH. **B** Western blotting with anti-His-tag monoclonal antibody. M, protein molecular weight marker, lane 1 and 2, whole cell lysate of *E. coli* BL21 carrying recombinant vector pET-28a-*gapdh* before and after induction by IPTG overnight; lane 3, purified rGAPDH through Ni-chelating affinity chromatography.

The GAPDH gene is very conserved among different strains of *M. hyorhini*s. Full-length of GAPDH gene, designed based on the genome sequence of strain HUB-1, was synthesized and cloned into the expression vector pET-28a ( +). The rGAPDH expressed in *E. coli* BL21 in soluble form was induced by IPTG and purified by nickel affinity chromatography. The purified rGAPDH was detected by SDS-PAGE as a band around 40 kDa, which corresponds to the size of GAPDH with a His tag (Figure 1A, lane 3). The rGAPDH was further identified by Western blotting with anti-His antibody (Figure 1B, lane 3). Anti-rGAPDH polyclonal antibody was prepared using the purified rGAPDH protein, with a titer of 1: 204 800 detected by ELISA. The antibody specifically reacted with the GAPDH of *M. hyorhinis* (Additional file 1).

### Surface localization of GAPDH in *Mycoplasma hyorhinis*

**Figure 2:**
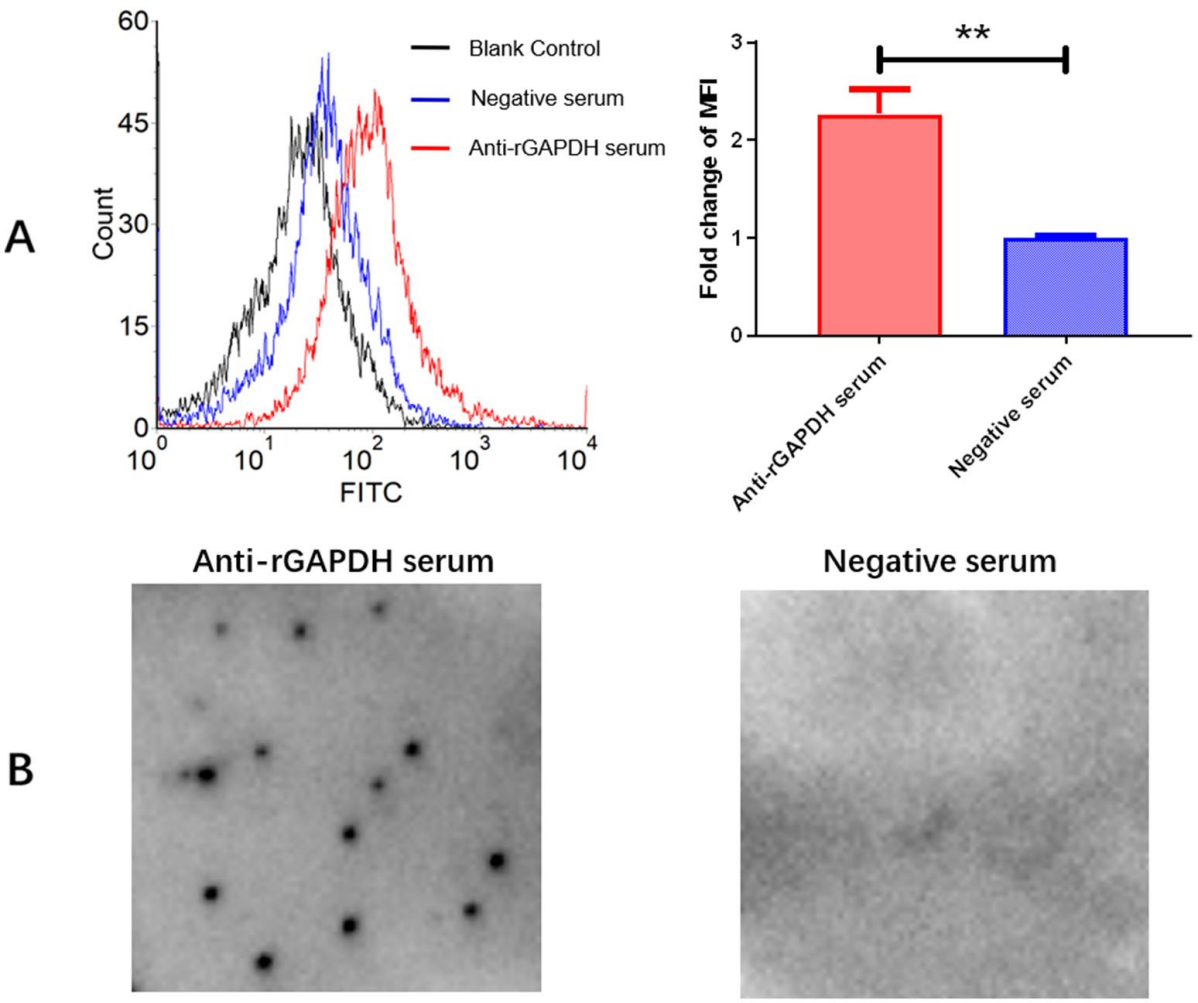
**Surface localization of GAPDH in *M. hyorhinis***. **A** Detection of GAPDH by flow cytometry. *M. hyorhinis* were incubated with anti-rGAPDH serum or negative serum respectively. *M. hyorhinis* treated with PBS was used as the blank control. The level of mean fluorescence intensity (MFI) of *M. hyorhinis* incubated with anti-rGAPDH serum is expressed as the percentage of the bacteria incubated with negative serum after deduction of the background (**P < 0.01). Data are expressed as mean ± SD of at least three experiments with samples in triplicate. **B** Detection of surface GAPDH by colony blot analysis. Reaction of *M. hyorhinis* colonies transferred to PVDF membrane was probed with anti-rGAPDH serum or negative serum.

To investigate the cell surface localization of GAPDH on *M. hyorhinis*, two approaches were used. A flow cytometry analysis showed a significantly higher fluorescence intensity (MFI) of the *M. hyorhinis* incubated with anti-rGAPDH serum than the *M. hyorhinis* incubated with the negative serum of the corresponding rabbits (Figure 2A), which indicated that the surface GAPDH was accessible to the GAPDH-specific antibody. To further confirm the surface localization of GAPDH, colony blot analysis was carried out with agar cultures incubated with anti-rGAPDH serum. The corresponding pre-immune serum was used as the negative control. The anti-rGAPDH serum reacted strongly with the *M. hyorhinis* colonies, whereas no signals were obtained after incubating with the negative serum (Figure 2B).

### Cytoadhesion of rGAPDH to host epithelial cells

**Figure 3:**
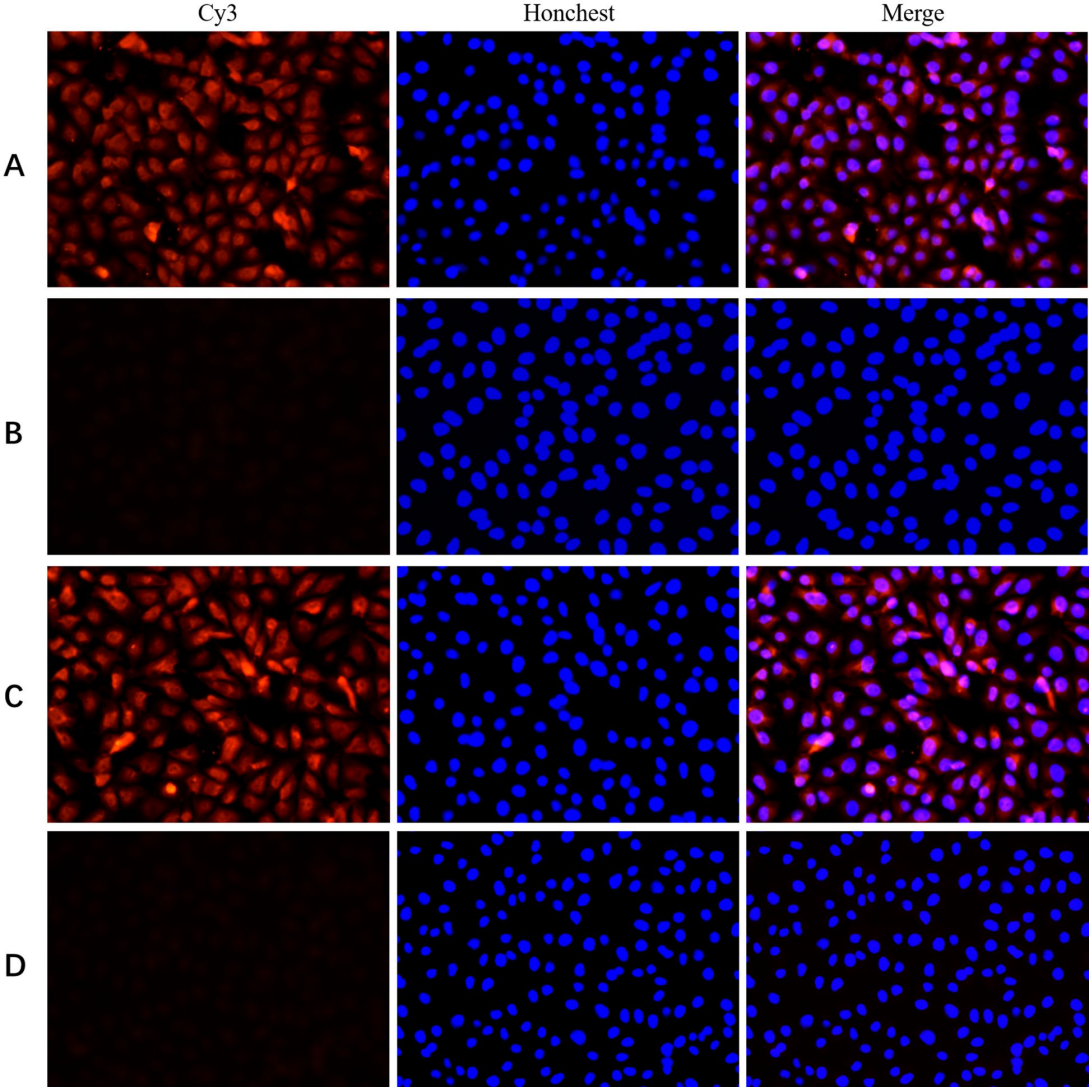
**Cytoadhesion of the rGAPDH protein detected by indirect immunofluorescence assay**. The rGAPDH (**A**, **C**) or BSA (**B**, D) was incubated with PK-15 (**A**, **B**) or NCI-H292 (**C**, **D**) cells, respectively. Bound protein was detected with anti-rGAPDH serum and Cy3-conjugated secondary antibody (red). Nuclei were stained with Honchest 33,342 (blue).

**Figure 4:**
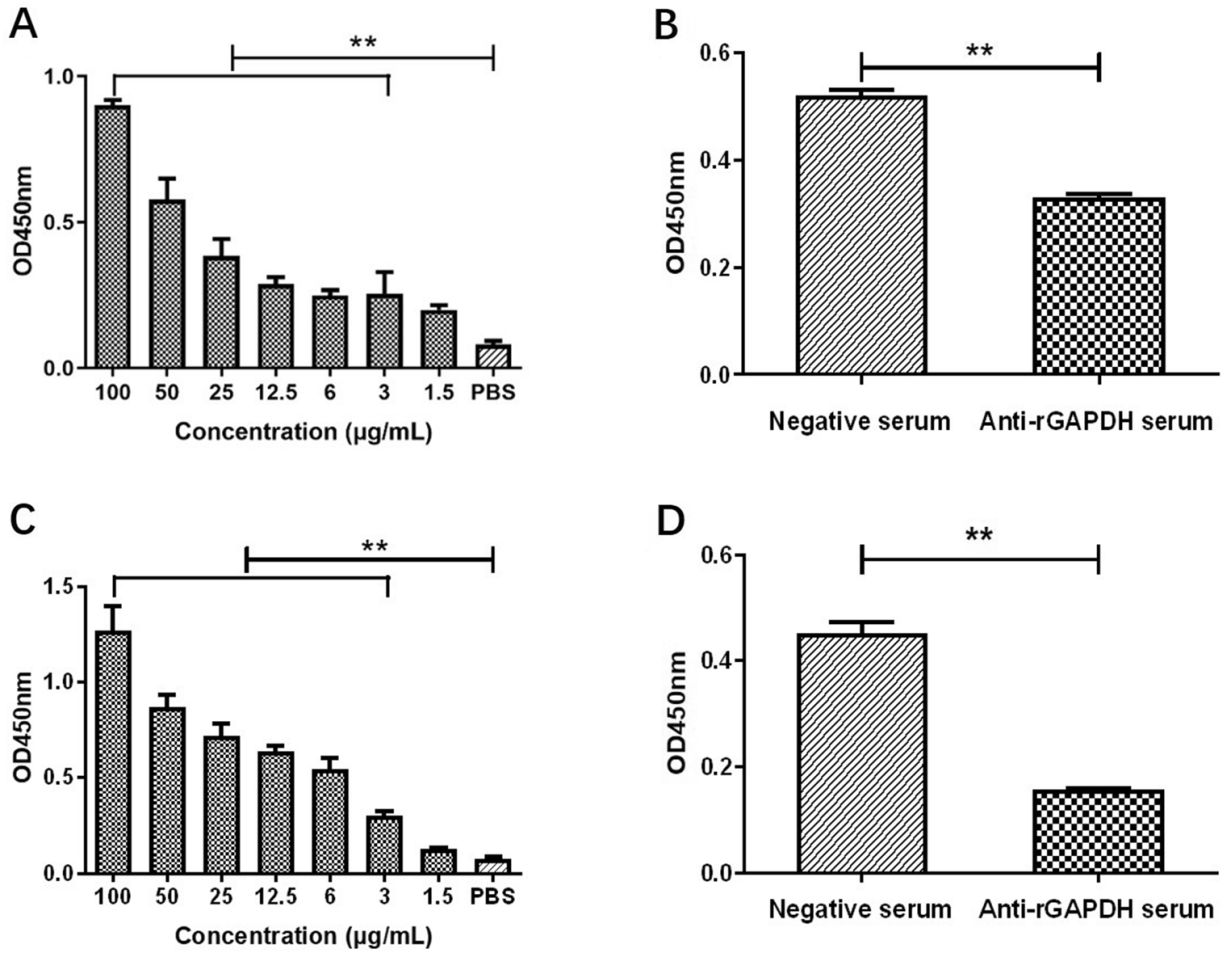
**Microtiter plate adhesion assay (MPAA) for the binding of the rGAPDH protein to cell membrane proteins**. Microtiter plates were coated with membrane protein of PK-15 or NCI-H292 cells. Increasing concentrations of rGAPDH protein were added to individual wells (**A**: PK-15; **C**: NCI-H292). Bound rGAPDH was detected with anti-His-tag monoclonal antibody compared with the wells with no added protein. The adhesion of 50 μg/mL of rGAPDH to the cells (**B**: PK-15; **D**: NCI-H292) was inhibited by anti-rGAPDH serum but not by the preimmune serum. Data are expressed as mean ± SD of at least three experiments with samples in triplicate. ** indicate *P* < 0.01.

rGAPDH protein was analyzed for adherence to swine PK15 cells and human NCI-H292 cells. The direct adhesion of rGAPDH to the cells was visualized by fluorescence microscopy. As shown in Figure 3, 100 μg of the rGAPDH indicated with red fluorescence significantly adhered to PK-15 and NCI-H292 cells (Figures 3A and C). No adherence was observed for the BSA control (Figures 3B and D). For adherence quantification, binding activity of the rGAPDH protein to the cells was assessed by micro titer plate adhesion assay (MPAA). Various dilutions of the rGAPDH solution were added to ELISA wells coated with cell membrane proteins. As shown in Figure 4A, the rGAPDH bound to PK-15 cell membrane proteins in a dose-dependent manner from 3 μg/mL to 100 μg/mL (*P* < 0.01). A similar pattern was observed when plates were coated with NCI-H292 cell membrane proteins (Figure 4C), from 3 μg/mL to 100 μg/mL (*P* < 0.01). The binding was inhibited by using antiserum against rGAPDH, but not the negative serum (Figures 4B and D).

### Inhibition of anti-rGAPDH serum on adhesion of *Mycoplasma hyorhinis* to host cells

**Figure 5:**
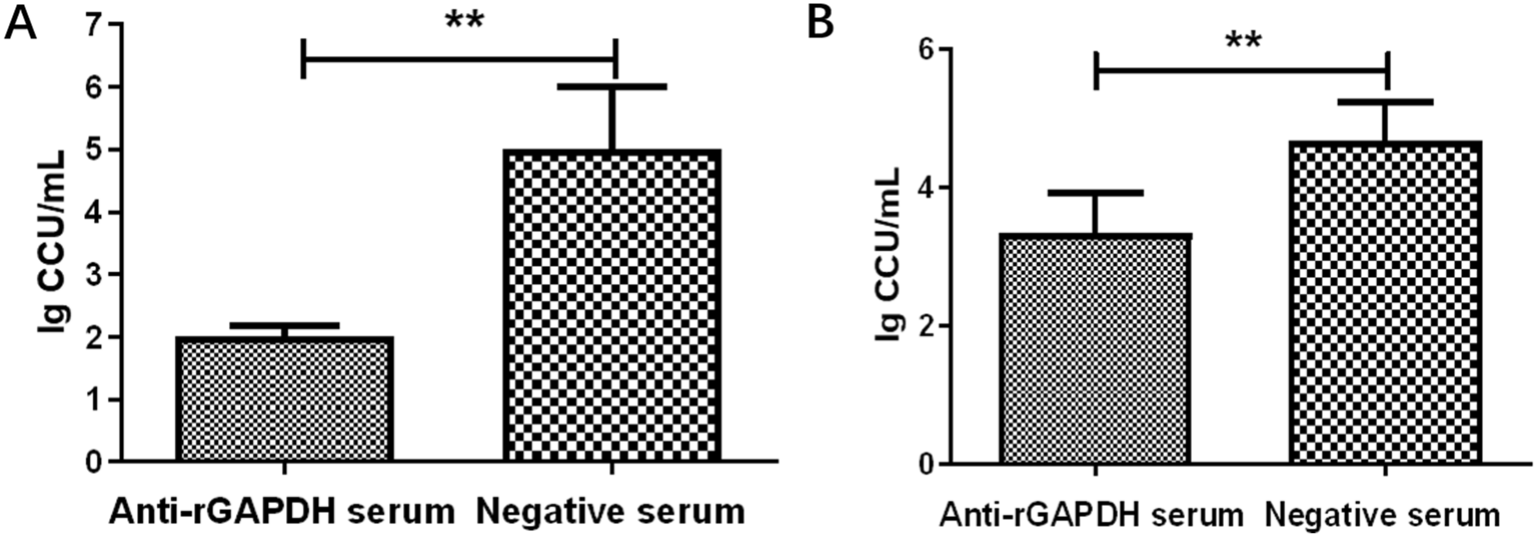
**Adhesion inhibition assay of anti-rGAPDH antibody**. *M. hyorhinis* were incubated with anti-rGAPDH serum or negative serum before adding into the cell culture plate. The number of mycoplasma adhering to the cells was expressed as CCU/mL. Data are expressed as mean ± SD of at least three experiments with samples in triplicate. **P < 0.01. **A** PK cell; **B** NCI-H292 cell.

Antibody inhibition assay was performed to further confirm the function of surface-exposed GAPDH in cytoadhesion of *M. hyorhinis*. The number of *M. hyorhinis* attached to PK15 Figure 5A) and NCI-H292 (Figure 5B) cells were greatly reduced after being incubated with anti-rGAPDH serum, compared with those of the *M. hyorhinis* incubated with negative serum (*P* < 0.01). It was confirmed that surface-localized GAPDH plays an indispensable role in adherence of *M. hyorhinis* to host cells.

### Binding activity of rGAPDH to plasminogen

**Figure 6:**
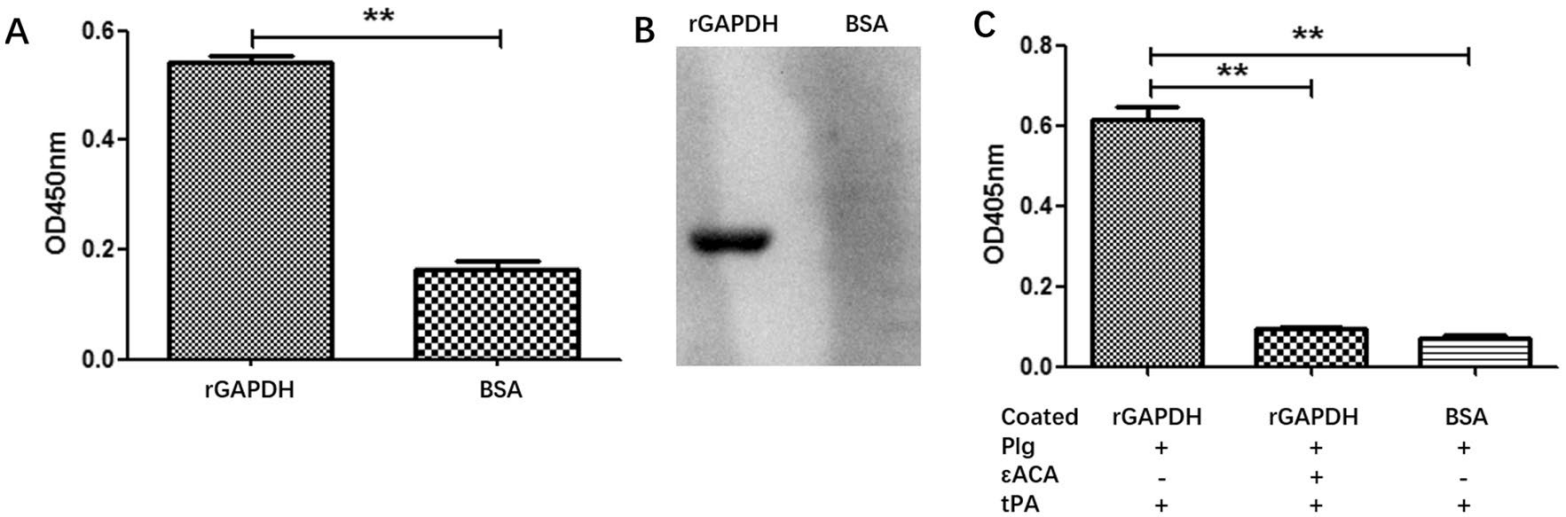
**Binding activity of the rGAPDH protein to plasminogen and the activation of the rGAPDH-bound plasminogen to plasmin**. **A** ELISA was performed to characterize the ability of immobilized rGAPDH binding plasminogen. **B** Far Western blot analysis of binding activity of rGAPDH to plasminogen. Bound plasminogen was determined by anti-plasminogen antibody. BSA was chosen as negative control for non-specific binding. **C** Plasminogen bound to the coated rGAPDH in microtiters plate was activated to plasmin by tPA. The presence of lysine analogue, ε-ACA decreased the binding of plasminogen to rGAPDH. Activity of plasmin was evaluated by adding chromogenic substrate, and OD405nm was measured. Data are expressed as mean ± SD of at least three experiments with samples in triplicate. **P < 0.01.

First, ELISA was used to define the ability of rGAPDH to bind plasminogen. Plasminogen was added into the micro titer plates coated with rGAPDH or BSA. After washing, bound plasminogen was detected with polyclonal anti-plasminogen. As shown in Figure 6A, the wells coated with rGAPDH shown significantly increased OD450nm values than those of the wells coated with BSA (*P* < 0.05), indicating a binding between rGAPDH and plasminogen. To further characterize the specific interaction between GAPDH and plasminogen, Far-Western bolt analysis was conducted. After rGAPDH or BSA was transferred to PVDF membrane, plasminogen was added. After washing, bound plasminogen could be detected with anti-plasminogen antibody at the site corresponding to the band of purified rGAPDH (Figure 6B). No obvious interaction between plasminogen and BSA was observed.

### Activation of the rGAPDH-bound plasminogen to plasmin

Plasminogen-plasmin system plays an important role in bacteria invasion. After confirmation of binding of rGAPDH to plasminogen, we further detected if rGAPDH-bound plasminogen could be activated to plasmin by the activator tPA. ELISA plates were coated with rGAPDH and blocked. After plasminogen and tPA were added successively, proteolytic activity was quantified using a plasmin-specific chromogenic substrate. As shown in Figure 6C, rGAPDH-bound plasminogen was converted to active plasmin by plasminogen activator tPA. The activity was detected with an OD 405 nm of 0.61, significantly higher than that of BSA-coated wells (*P* < 0.01) and that of wells in the presence of the lysine analogue, ε-ACA (*P* < 0.01), which not only indicated that rGAPDH-bound plasminogen can be activated but also hinted that plasminogen probably binds to rGAPDH through the lysine residues of the latter.

### Degradation of a reconstituted basement membrane by the rGAPDH-bound plasmin

**Figure 7:**
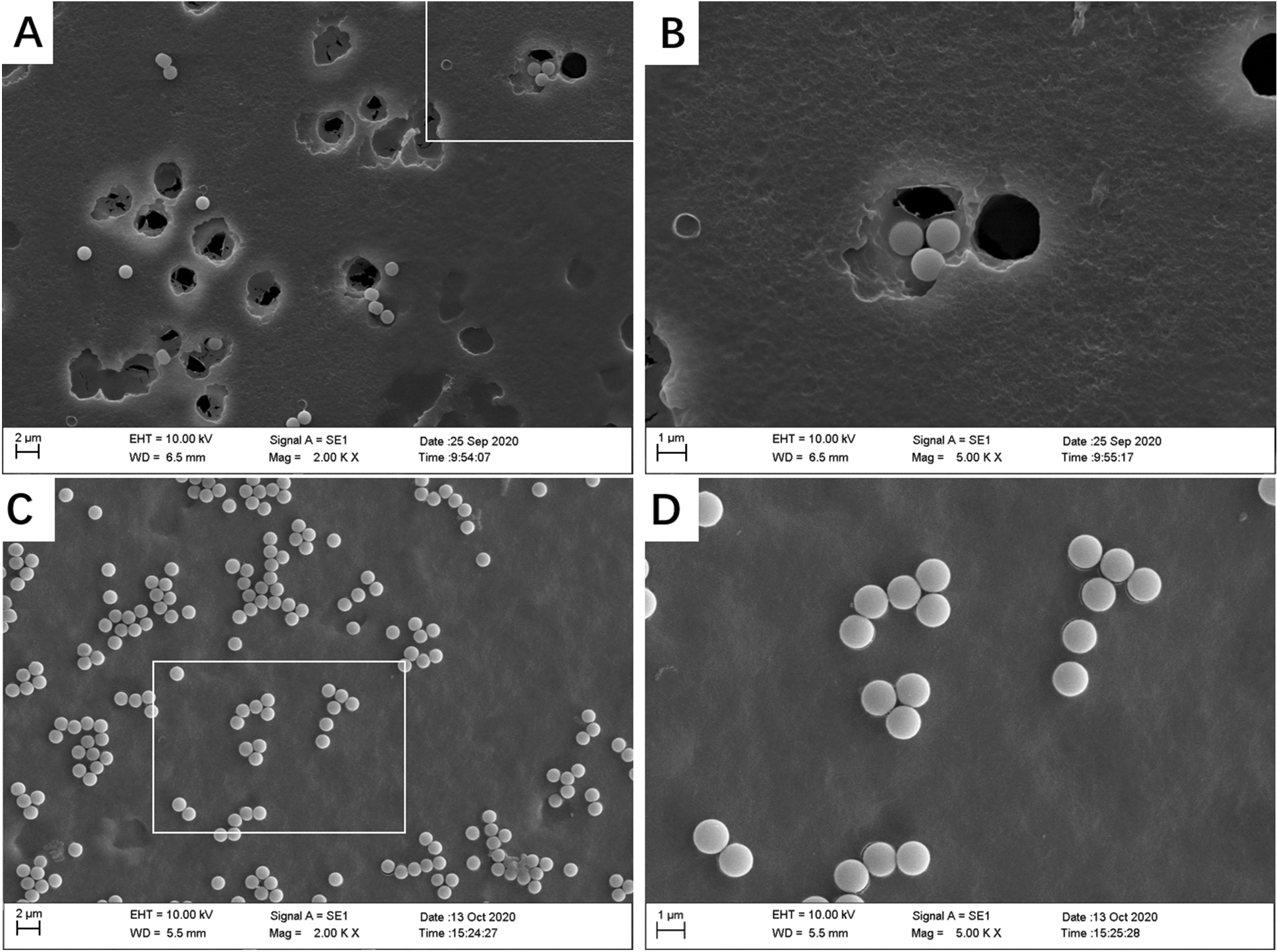
**Electron microscopic visualization of the degradation of Matrigel reconstituted basement membrane**. The rGAPDH-harboring polystyrene beads (**A**, **B**) or the BSA-harboring beads (**C**, **D**) were incubated with plasminogen and tPA, and then added on the 3-μm filters in Transwell cell culture chamber inserts previously coated with Matrigel reconstituted basement membrane. After an incubation of 40 h, the filters were fixed with 2.5% glutaraldehyde, and examined in scanning electron microscope. **B** and **D** are the enlarged view of a part of **A** and **C**, respectively.

ECM degradation is critical in the invasion process of bacteria. The basement membrane under the epithelial cells is assumed to be one of the most important physical barriers that restrict *M. hyorhinis* from breaking through the respiratory tract. We further performed the proteolytic activity test on Matrigel, a complex ECM preparation composed of laminin, type IV collagen, heparansulfate proteoglycan, etc. Basement membrane was reconstituted by Matrigel on the 3-μm filters in Transwell cell culture chamber inserts. rGAPDH or BSA-coated polystyrene beads treated with plasminogen and tPA were added. The degradation of reconstituted basement membrane was analyzed by electron microscopy. The structure of the reconstituted basement membrane is shown in Figure 7. With the supplementation of plasminogen and tPA, no degradation was observed for the BSA-coated beads (Figures 7C, D). While significant damage of the reconstituted basement membrane, forming depressions emerged in the in vitro model of ECM membrane treated with rGAPDH-coated beads. The holes in the filter membrane were exposed in some area, where the beads can fall into (Figures 7A, B).

### Binding activity of rGAPDH to ECM components

**Figure 8:**
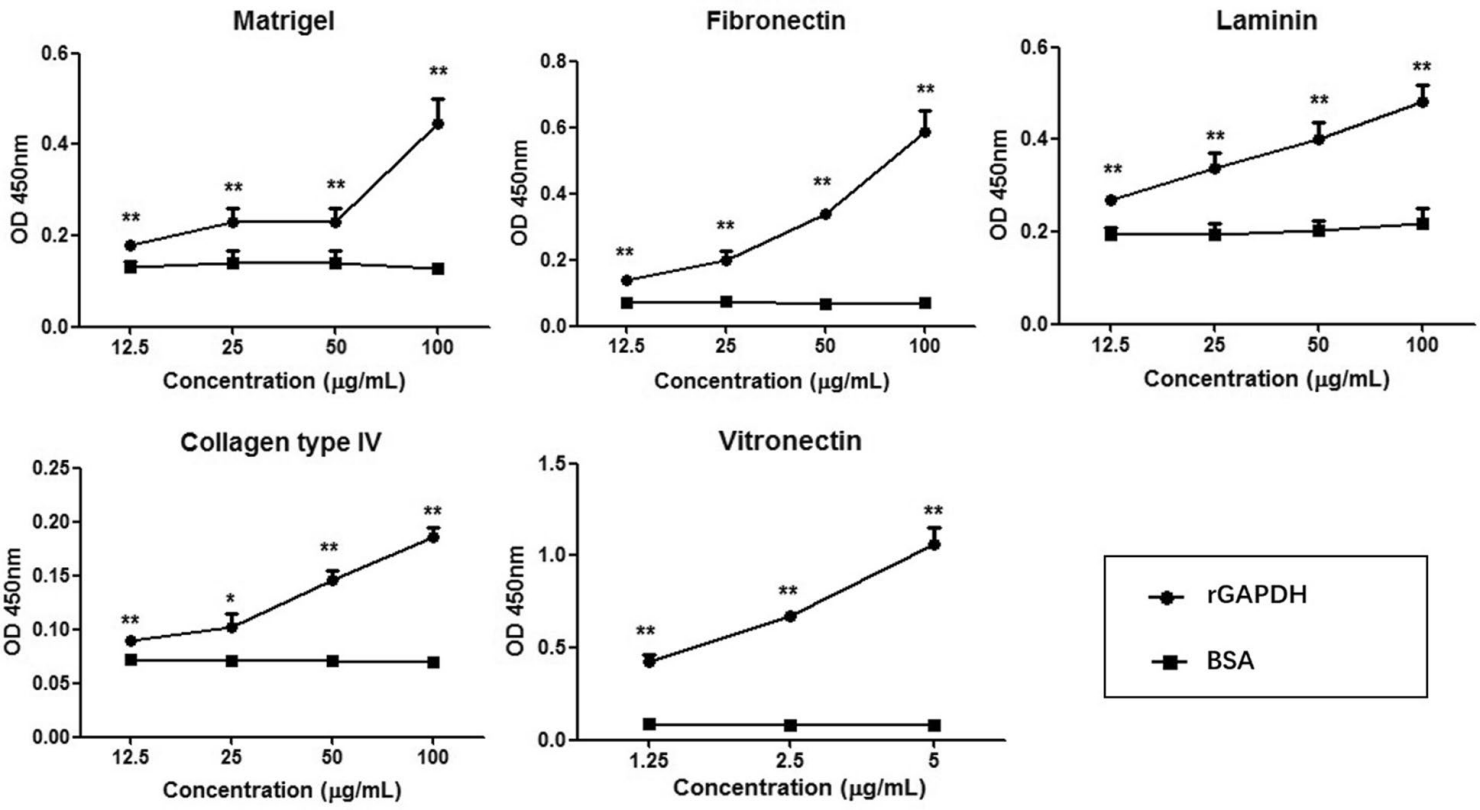
**Binding of the rGAPDH protein to different ECM components in ELISA experiment**. Microtiters plate was coated with Matrigel, fibronectin, collagen or laminin solution. Various concentrations of rGAPDH or BSA were added and detected by anti-His-tag monoclonal antibody. For detecting the binding to vitronectin, microtiters plate was coated with rGAPDH or BSA. Various concentrations of vitronectin were added and detected by anti-vitronectin monoclonal antibody. *P < 0.05, **P < 0.01, compared with the negative control (BSA).

ECM components are common host proteins that interact with mycoplasmas. The combination of mycoplasmas to ECM facilitates the subsequent degradation. Here, we evaluated the interaction between rGAPDH and different ECM components to further study which component of ECM is the main receptor for GAPDH binding. By coating Matrigel, fibronectin, laminin, and collagen type IV on micro titer plates (the system was inverted for detecting the binding of rGAPDH to recombinant vitronectin by coating rGAPDH on wells), concentration-dependent binding of rGAPDH to Matrigel and all of the four ECM components were observed (*P* < 0.01). The binding of rGAPDH with ECM is multiple guaranteed (Figure 8).

## Discussion

*Mycoplasma hyorhinis* is commonly found in pigs, especially in the respiratory tract. The occurrence of disease is often related to the systemic invasion of the pathogen. *M. hyorhinis* has also been detected in a variety of human tumor tissues, such as gastric, esophageal, lung, breast, glioma and colon cancers [2]. Adhesion to the host cell is the first step in many mycoplasmas infection. Most mycoplasmas lack the classical bacterial toxins; therefore, adhesion molecules are considered to be important virulence-related factors for mycoplasmas, and are often regarded as the main candidate antigens for vaccine development. To date, the Vlp family and P37 proteins are the well-known identified adhesins of *M. hyorhinis*, the cytoadhesive function of which have been proven in swine respiratory epithelium cells [6] and human gastric cancer cells [7]. However, the pathogenesis and possible virulence factors of *M. hyorhinis* are not yet fully known, and the exact mechanism by which it adheres to epithelial cells remain to be understood. In the present study, we demonstrated a novel adhesin of *M. hyorhinis*, GAPDH. GAPDH is originally one of the key canonical glycolytic enzymes, which functions in the cytoplasm. However, it can also be present at the surface of some pathogens, where it plays other roles, including acting as an adhesion molecule, although it lacks special extracellular targeting motifs [29–31]. The surface location and cytoadhesion function of GAPDH have been reported in three *Mycoplasma* species, *M. pneumoniae* [12, 32], *M. hyopneumoniae* [18] and *Mycoplasma suis* [33, 34]. The present study focuses on GAPDH’s role as a non-canonical adhesin in *M. hyorhinis*. The cell-surface display of GAPDH in *M. hyorhinis* was determined by flow cytometry and colony blot analyses. This suggested that GAPDH has the possibility to interact directly with host cells or components. Furthermore, we demonstrated the adhesion of the GAPDH of *M. hyorhinis* to two commonly used cell lines, porcine-derived PK-15 cells and human-derived NCI-H292 cells by recombinant protein adhesion and antibody inhibition tests. The data indicated that the GAPDH moonlights as an adhesin on the *M. hyorhinis* surface. However, in the competitive adhesion inhibition assay, only partial inhibition of GAPDH was observed. Since several other proteins, such as P37 and Vlp have also been verified to contribute to the adhesion process of *M. hyorhinis*, it can be speculated that when GAPDH is blocked, the adhesion of *M. hyorhinis* still partly exists.

*Mycoplasma hyorhinis* is considered to be commensal, colonizing the tonsils and respiratory epithelium of the nasal cavity and conducting airways. The occurrence of disease is often related to the systemic invasion of *M. hyorhinis.* In addition to tonsil, nasal cavity and lung, *M. hyorhinis* could also be detected or recovered from a variety of other tissues from the infected pigs with clinical symptoms, including heart, pericardium, peritoneum, pericardial fluid, peritoneal fluid, affected joints, spleen, liver, inguinal node, and even from central nervous system [23, 35–38]. The detection of bacterium in samples collected from affected joints and/or serous membranes is generally required for accurate diagnosis [1]. However, the mechanism of its dissemination is unknown. The plasminogen/plasmin system of the host plays a significant role in several physiological processes, such as degradation of fibrin clots in fibrinolysis and various ECM and connective tissue components, such as laminin and fibronectin [39, 40]. Many bacteria interact with the plasminogen/plasmin system by secreting PAs or, more commonly, by expressing PlgRs on their surface. Binding of plasminogen to bacterial PlgR enhances plasminogen activation by host PAs, and the bacteria consequently turn themselves into proteolytic organisms using the host-derived system [24, 25]. The ability to degrade tissue barriers formed by the ECM, leading to tissue and structure damage, is one of the most important factors in bacterial invasiveness of the host body.

Bacteria usually employ multifunctional, highly conserved proteins to capture plasminogen. Members of the glycolytic pathway, chaperones, and other metabolic enzymes have been identified as plasminogen-binding proteins from diverse bacteria [41]. Enolase, a key glycolytic enzyme, is one of the most well-known PlgRs [42, 43]. In addition, GAPDH is another widely studied PlgR. Its plasminogen binding function has been reported in many bacteria, such as streptococci [44, 45], *Escherichia coli* [46], *Clostridium perfringens* [47], and *Erysipelothrix rhusiopathiae* [30] as well as certain parasites, such as *Babesia microti* [48] and *Dirofilaria immitis* [49], and even mammalian cells [50]. Among *Mycoplasma* species, the GAPDH of *M. pneumoniae* is the most studied. Different reports have confirmed that *M. pneumoniae* GAPDH can bind to plasminogen [12, 16, 20, 32]. However, the activity of the converted plasmin, especially its ability to degrade the ECM, was not significant or consistent in most of the published studies [16, 20]. In the present study, we demonstrated that rGAPDH of *M. hyorhinis* could bind plasminogen, and the bound plasminogen could be activated by tPA to form the active serine protease plasmin and degrade specific substrates. The binding and activity were markedly inhibited by adding εACA enriched in lysine, indicating the critical role of lysine residues in the interaction between GAPDH and plasminogen. The activity of produced plasmin was further demonstrated using Matrigel degradation analysis. These results indicated the role of the surface located GAPDH as a PlgR. To the best of our knowledge, this is the first demonstration of reconstituted ECM degradation by plasmin in the presence of GAPDH of mycoplasma. Systemic infection is critical for *M. hyorhinis* to cause disease; therefore, the adhesion molecules that also participate in the plasminogen/plasmin system activation should have more potential as subunit vaccine antigens.

According to current reports, *M. hyorhinis* has been detected in a variety of tumor tissues, such as gastric cancer and prostate cancer [5, 6]. It is likely that it would disseminate in the human body to form a systemic infection, as it does in pigs [1, 23, 35–38,], and the exploitation of the plasminogen/plasmin system might also participate in its infection in humans. On the other hand, dysregulation of the plasminogen/plasmin system is also involved in tumor growth and metastasis formation [51, 52]. Indeed, overexpression of PlgRs, including actin, enolase-1, cytokeratin 8, and annexin II-S100A10, has been associated with poor prognosis and resistance to chemotherapy in patients with cancer [53]. A number of experiments have shown that *M. hyorhinis* infection can induce a variety of tumor cells to increase their migration and invasiveness in vitro [54–56]. Some explanations have been proposed, including the key function of P37 [57, 58]; however, the precise mechanism by which P37 functions remains unknown. A preliminary test has been carried out, which showed that the amount of plasminogen bound to the surface of *M. hyorhinis*-infected NCI-H292 cells was higher than that of the uninfected cells [data not shown]. This suggested that *M. hyorhinis* might act as a bridge to enhance the ability of tumor cells to capture plasminogen to their surface, thereby affecting the subsequent tumor development process.

In addition to binding plasminogen, GAPDH has a variety of other binding activities. The GAPDHs of *Clostridium perfringens* [47] and *Erysipelothrix rhusiopathiae* [30] can also bind to fibronectin. The interaction between *M. pneumoniae* GAPDH and different ECM components [16, 32], as well as the interaction between *Mycoplasma genitalium* GAPDH and mucin [59], have also been reported. Here, we demonstrated the interaction between rGAPDH of *M. hyorhinis* and fibronectin, laminin, collagen type IV, and vitronectin. Speculatively, in the process of infection, *M. hyorhini*s first uses adhesion factors such as GAPDH to bind the ECM, facilitating adhesion and colonization. It then recruits plasminogen, and uses activated plasmin to degrade the ECM to help it spread through tissue barriers. The characteristic of one receptor binding to multiple host molecules could help to efficiently direct the plasmin activity to locations where proteolytic activity is required.

GAPDH is a conserved house-keeping enzyme. Among the *Mycoplasma* species that infect swine, the amino acid sequence identity of the GAPDH protein of *M. hyorhinis*, *M. hyopneumoniae*, and *Mycoplasma flocculare* is above 73%, and the identity between *M. hyorhinis* and *Mycoplasma hyosynoviae* is 58.51%. The surface exposure of the GAPDH protein of *M. hyopneumoniae*, as well as its ability to bind actin, fibronectin, heparin and plasminogen, have been reported [18]. Recently, we demonstrated the hydrolytic activity of its bound plasminogen after activation (non-published results). We speculated that these functions of GAPDH might be universal in swine mycoplasmas.

Present study demonstrated that GAPDH acts as a surface adhesin in *M. hyorhinis* adhesion to porcine PK-15 cell and human NCI-H292 cell. It also acts as a receptor in the interaction between *M. hyorhinis* and plasminogen. The activation of the rGAPDH-bound plasminogen has been demonstrated, which resulted in ECM degradation. The binding of rGAPDH to different ECM components has also been proved, and it could help to direct the plasmin activity to locations required. Possible lysine residues binding sites were raised in this study which was worthy to be studied further.

## Supplementary Information

**Additional file 1: Assessment of the reactivity and specificity of the prepared polyclonal antibody against rGAPDH**. **A** The whole cell protein of *E. coli* and purified rGAPDH protein were subjected to 12% SDS-PAGE and transferred to a polyvinylidene fluoride (PVDF) membrane. After blocking with 5% skim milk in TBST buffer, the membrane was incubated with the anti-GAPDH sera (1:5000), followed by horseradish peroxidase (HRP)-conjugated goat anti-rabbit IgG (1:10 000 dilution). Finally, filters were developed with Electro-Chemi-Luminescence (ECL) substrate using a ChemiDoc XRS + system (Bio-Rad, USA). M, protein molecular weight marker, lane 1, whole cell lysate of *E. coli* BL21 carrying empty vector pET-28a( +) before induction, lane 2 and 3, whole cell lysate of *E. coli* BL21 carrying recombinant vector pET-28a*-gapdh* before and after induction by IPTG overnight; lane 4, purified rGAPDH through Ni-chelating affinity chromatography. **B**, **C** The hybridization to the whole cell lysate of *M. hyorhinis* was furtherly conducted to assess the specificity of the polyclonal antibody against rGAPDH (**B**). The sera obtained before immunization was used as negative control (**C**). M, protein molecular weight marker; lane 1, whole cell lysate of *M. hyorhinis*, lane 2, purified rGAPDH.

## Abbreviations

BSA: Bovine serum albumin
BM: Basement membrane
CCU: Color change unit
ELISA: Enzyme-linked immunosorbent assay
ECM: Extracellular matrix
GAPDH: Glyceraldehyde-3-Phosphate Dehydrogenase
MFI: Mean fluorescence intensity
MPAA: Micro titer plate adhesion assay
PlgR: Plasminogen receptor
PA: Plasminogen activator
PCR: Polymerase chain reaction
rGAPDH: Recombinant Glyceraldehyde-3-Phosphate Dehydrogenase
tPA: Tissue plasminogen activator
uPA: Urokinase plasminogen activator
Vlp: Variable lipoprotein
ε-ACA: ε-Aminocaproic acid
