# Supplementary figures and images for: Glyceraldehyde-3-phosphate dehydrogenase (GAPDH) moonlights as an adhesin in Mycoplasma hyorhinis adhesion to epithelial cells as well as a plasminogen receptor mediating extracellular matrix degradation
Source: Vet Res. 2021 Jun 3;52:80. doi: 10.1186/s13567-021-00952-8 (PMC8173509; doi:10.1186/s13567-021-00952-8)

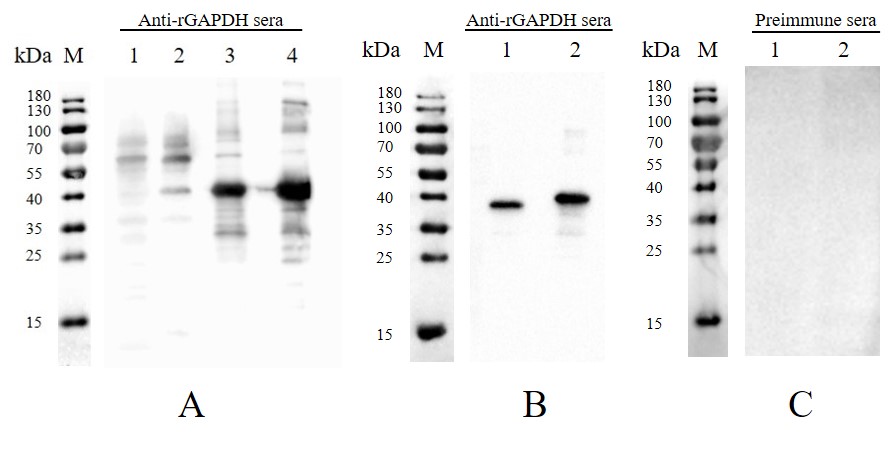

Supplement: Supplementary file 1 — Additional file 1: Assessment of the reactivity and specificity of the prepared polyclonal antibody against rGAPDH. A The whole cell protein of E. coli and purified rGAPDH protein were subjected to 12% SDS-PAGE and transferred to a polyvinylidene fluoride (PVDF) membrane. After blocking with 5% skim milk in TBST buffer, the membrane was incubated with the anti-GAPDH sera (1:5000), followed by horseradish peroxidase (HRP)-conjugated goat anti-rabbit IgG (1:10 000 dilution). Finally, filters were developed with Electro-Chemi-Luminescence (ECL) substrate using a ChemiDoc XRS + system (Bio-Rad, USA). M, protein molecular weight marker, lane 1, whole cell lysate of E. coli BL21 carrying empty vector pET-28a( +) before induction, lane 2 and 3, whole cell lysate of E. coli BL21 carrying recombinant vector pET-28a-gapdh before and after induction by IPTG overnight; lane 4, purified rGAPDH through Ni-chelating affinity chromatography. B, C The hybridization to the whole cell lysate of M. hyorhinis was furtherly conducted to assess the specificity of the polyclonal antibody against rGAPDH (B). The sera obtained before immunization was used as negative control (C). M, protein molecular weight marker; lane 1, whole cell lysate of M. hyorhinis, lane 2, purified rGAPDH. [file 13567_2021_952_MOESM1_ESM.docx]
